# Population aging and endogenous economic growth

**DOI:** 10.1007/s00148-012-0441-9

**Published:** 2012-09-15

**Authors:** Klaus Prettner

**Affiliations:** 1 Center for Population and Development Studies, Harvard University, 9 Bow Street, Cambridge, MA 02138 USA; 2 Institute for International Economics, University of Göttingen, Platz der Göttinger Sieben 3, 37073 Göttingen, Germany

**Keywords:** Demographic change, Technological progress, Long-run economic growth

## Abstract

We investigate the consequences of population aging for long-run economic growth perspectives. Our framework incorporates endogenous growth models and semi-endogenous growth models as special cases. We show that (1) increases in longevity have a positive impact on per capita output growth, (2) decreases in fertility have a negative impact on per capita output growth, (3) the positive longevity effect dominates the negative fertility effect in case of the endogenous growth framework, and (4) population aging fosters long-run growth in the endogenous growth framework, while its effect depends on the relative change between fertility and mortality in the semi-endogenous growth framework.

**Electronic supplementary material** The online version of this article (doi:10.1007/s00148-012-0441-9) contains supplementary material, which is available to authorized users.

## Introduction

Population aging in industrialized countries has been identified as a central topic regarding future economic development. It has gained attention in academic research as well as in the public debate (see, for example, Bloom et al. 2008, 2010a, 2011; The Economist 2009, 2011, for an overview). While declining fertility—even below the replacement level—triggers increases in the mean age of a certain population and slows down population growth, decreasing old age mortality allows individuals to enjoy the benefits of retirement for longer time periods. Just to get an impression of the severity of the demographic changes, we are facing the following: on the global scale, the total fertility rate has dropped from five children per woman in 1950 to 2.5 children per woman today, while life expectancy has increased from 48 years in 1950 to 68 years today (cf. United Nations 2011; Bloom et al. 2011). The economic consequences of these developments are expected to be huge. To mention only the most well known examples, support ratios will decline such that fewer and fewer workers will have to carry the burden of financing more and more retirees (see for example Gertler 1999; Gruescu 2007); overall productivity levels will change because workers have age-specific productivity profiles and the age decompositions of societies will shift (see Skirbekk 2008, for an overview); and the savings behavior of individuals will change because they expect to live longer (see for example Heijdra and Ligthart 2006; Heijdra and Romp 2008).

However, as regards the implications of population aging for per capita output growth in a setting with diminishing marginal products of capital, there are only *transient* effects of changing support ratios, changing savings behavior of households, and changing aggregate productivity profiles. The reason is that a shift from high to low fertility cannot lead to a *permanently changing* age decomposition of a certain population (cf. Preston et al. 2001), and the induced change in the savings behavior of households has only *level* effects on per capita output (cf. Solow 1956; Cass 1965; Koopmans 1965; Diamond 1965). By contrast, we are interested in the implications of population aging for per capita output *growth* over a *long* time horizon. Since technological progress has been identified as the main determinant of long-run economic prosperity (see for example Romer 1990; Aghion and Howitt 1992; Jones 1995a; Segerström 1998), we are particularly concerned with the effects of changing age decompositions on research and development (R&D) intensities. The natural model classes to investigate these effects are endogenous and semi-endogenous growth frameworks, where the R&D effort of a society is determined by general equilibrium forces due to the interaction between utility-maximizing households and profit-maximizing firms.

Endogenous growth models with purposeful R&D investments (see for example Romer 1990; Grossman and Helpman 1991; Aghion and Howitt 1992) state that, aside from other influences, the population size of a certain country is crucial for its long-run economic development. The argument is that larger countries are able to grow faster because they have more scientists to employ and feature larger markets with more profit opportunities for innovative firms. This is called the scale effect, which was, however, questioned by Jones (1995b) because it had not been supported by empirical evidence. In another contribution, Jones (1995a) paved the way for semi-endogenous growth models (see also Kortum 1997; Segerström 1998), where long-run economic performance is affected by population *growth* rather than population *size*. The basic idea of semi-endogenous growth models is that developing a constant share of new technologies becomes more and more difficult with an expanding technological frontier. Consequently, ever more scientists have to be devoted to R&D activities in order to sustain a certain pace of technological progress. In the long run, this can only be achieved by having positive population growth.

Despite that the described models examine the economic growth effects of changes in demographic patterns as represented by population size and population growth, they remain silent when it comes to the consequences of population aging. The reason is their common underlying assumption that economies are populated by representative *identical* individuals who live forever. We introduce age-specific heterogeneity of individuals by generalizing these frameworks to account for finite individual planning horizons and overlapping generations in the spirit of Blanchard (1985) in case of the endogenous growth paradigm and in the spirit of Buiter (1988) in case of the semi-endogenous growth paradigm. In so doing, we assume that individuals do not live forever but that they have to face a certain probability of death at each instant. Furthermore, we allow for endogenous fertility choices inspired by Barro and Becker (1989), Sato and Yamamoto (2005), and Miyazawa (2006), where parents want to have children, while they also face the associated costs in terms of foregone consumption. The standard endogenous and semi-endogenous growth models are then special cases of our framework with the probability of death being equal to zero and fertility being exogenously given.

For analytical tractability, the mortality rate has to be age-independent in our model. Consequently, the interactions between child mortality and fertility decisions cannot be studied satisfactorily within its realm. Nevertheless, we acknowledge that this is a very important topic: Doepke (2005) analyzes the implications of decreasing child mortality for the predictions of the Barro and Becker (1989) model and concludes that a decrease in child mortality alone cannot explain the slowdown of population growth within this framework. By contrast, Kalemli-Ozcan (2002, 2003) assume that parents are risk averse and use this framework to investigate the interactions between child mortality and human capital investments. The finding is that a decline in the exogenous child mortality rate reduces precautionary demand for children, which has the potential to reduce population growth. The slowdown in population growth, in turn, allows parents to raise investments in children’s human capital, which enables countries in a preindustrial stage of development to escape from the Malthusian trap. Furthermore, Cigno (1998) focuses on the implications that endogenous child mortality has on the fertility decision of parents and finds that mortality and fertility are positively related if parents realize that they can increase the survival probabilities of their children by spending more for child nutrition and health. If child mortality is high (less developed countries), governmental investments aimed at reducing child mortality raise parent’s investments in the number of children as well as in the nutrition and health of these children, which altogether promotes population growth. By contrast, if child mortality is low (developed countries), further governmental investments in reducing child mortality mainly lead to lower investments of parents both in the number of their kids as well as in their nutrition and health. Thus, at this stage of development, governmental policies that reduce child mortality primarily represent a subsidy on parent’s consumption. We conclude that very interesting and relevant interactions occur once that (endogenous) child mortality is taken into account. It should therefore be considered in a follow-up simulation study, where analytical tractability is less important and therefore mortality can be allowed to vary with age.

Altogether, our results indicate that a more realistic demographic structure in traditional endogenous and semi-endogenous growth models is desirable because it allows to disentangle the growth effects of a changing population size from those of a changing population age structure. We can also show that the growth effects of population aging differ between the endogenous growth paradigm and the semi-endogenous growth paradigm. In particular, we find that decreasing mortality has a positive effect on long-run growth, while the converse holds true for decreasing fertility. The positive effects of decreases in mortality outweigh the negative effects of decreases in fertility in case of the Romer (1990) model, while the positive and negative effects exactly offset each other in the Jones (1995a) framework. Furthermore, population aging positively impacts long-run economic growth in the Romer (1990) case, whereas its particular effect in the Jones (1995a) case depends on the relative change between fertility and mortality.

Two other branches of the literature closely relate to our efforts. The first one (Reinhart 1999; Futagami and Nakajima 2001; Petrucci 2002) basically follows the Romer (1986) assumption that there are knowledge spillovers in the production process, and, hence, there are no diminishing returns of capital in the aggregate production function. This assumption allows them to draw conclusions on the effects of demographically induced changes in individual savings behavior even on long-run economic growth performance. A very interesting recent contribution (Schneider and Winkler 2010) uses this framework to endogenize the rate of mortality and to analyze the welfare implications of individual health investments. However, the knowledge spillover model of Romer (1986) has been criticized because empirical evidence rather points toward a diminishing marginal product of capital (cf. Mankiw et al. 1992). Furthermore, one cannot analyze the effects of aging on purposeful R&D investments within such a framework and, as we will see later on, the transmission mechanism of the impact of aging on economic growth within these types of models differs to our approach because we also allow for an endogenous interest rate.

The second related branch to our work (Kalemli-Ozcan et al. 2000; Cervellati and Sunde 2005; Hazan and Zoabi 2006) focuses on the implications of population aging on human capital accumulation and basically states that an increase in the life expectancy of individuals renders investments in human capital more profitable. Consequently, human capital accumulation increases, which fosters economic growth via the particular link that these models establish between human capital accumulation and economic development.^1^ However, also these models do not consider the effects of aging on purposeful R&D investments and therefore the transmission mechanism of the effects of aging on economic growth is, by its very nature, different to ours.

The paper proceeds as follows: Section 2 describes a model that nests the Romer (1990) and the Jones (1995a) frameworks as special cases and features a richer demographic structure. Section 3 examines the effects of demographic change for long-run economic growth perspectives in the nested specifications. Section 4 draws conclusions and highlights scope for further research.

## The model

This section characterizes the basic model of R&D-based economic growth with overlapping generations and endogenous fertility. It nests the Romer (1990) framework with strong spillovers in the research sector and a constant population size and the Jones (1995a) framework with weaker spillovers in the research sector and a growing population size as special cases (cf. Strulik 2009).

### Basic assumptions and their implications

The basic structure of our model economy is that there are three sectors: final goods production, intermediate goods production, and R&D. The economy has two productive factors at its disposal: capital and labor. Labor and machines are used to produce final goods for a perfectly competitive market, capital and blueprints are used in the Dixit and Stiglitz (1977) monopolistically competitive intermediate goods sector to produce machines, and labor is used to produce the machine-specific blueprints in the R&D sector. Note that the R&D sector competes for labor with the final goods sector on the perfectly competitive labor market.

Technological progress is considered to be horizontal, that is, it is represented by the introduction of new product varieties in the intermediate goods sector. Using a model with vertical innovations by assuming that technological progress takes the form of quality improvements of existing product varieties would not change our qualitative results with respect to the long-run growth rate. The reason is that the drivers of long-run growth in the corresponding endogenous growth literature (Grossman and Helpman 1991; Aghion and Howitt 1992) and semi-endogenous growth literature (Segerström 1998) are qualitatively very similar to the generic models of horizontal innovation. However, while the long-run growth rate in models of horizontal innovation is always too low from a social perspective, this is not true for models of vertical innovation. In these frameworks, a quality improvement of an existing product by a contesting firm drives the incumbent out of business. This so called “business stealing effect” reduces the profits of established firms and therefore has a negative impact on overall welfare. Consequently, the possibility arises that—from a social perspective—innovation could be too fast and long-run economic growth too high. This means that the welfare effects of demographic change might be different if models of vertical innovations were considered as baseline frameworks in our study.^2^

In contrast to the representative agent assumption on which the Romer (1990) and Jones (1995a) frameworks rely, we introduce overlapping generations in the spirit of Blanchard (1985) to the Romer (1990) case and in the spirit of Buiter (1988) to the Jones (1995a) case.^3^ We assume that the population of an economy consists of different cohorts that are distinguishable by their date of birth denoted as *t*_0_. Each cohort consists of a measure *N*(*t*_0_, *t*) of individuals at a certain instant *t* > *t*_0_. In addition, individuals have to face a constant age-independent instantaneous risk of death which we denote by *μ*. Due to the law of large numbers, this expression also refers to the fraction of individuals dying at each instant. Furthermore, we endogenize fertility decisions of individuals in the sense that their utility increases in the number of children they have, while raising children requires parents to forego consumption (cf. Barro and Becker 1989; Sato and Yamamoto 2005; Miyazawa 2006).^4^ The fertility decisions of individuals allow for population growth, population stagnation and population decline, depending on the level of mortality. We will examine the growth effects of all these possible outcomes for both specifications of the underlying R&D-based economic growth model. Since the long-run growth rates tended to be constant in industrialized countries over the last decades (cf. Kaldor 1957; Jones 1995a; Acemoglu 2009), we will, in the next step, focus our attention on balanced growth paths (BGPs). By its definition, a BGP implies constant long-run growth, which requires the population to stay constant in the Romer (1990) framework and to grow in the Jones (1995a) framework. In our framework, both cases are possible outcomes for different parameter specifications with respect to the fertility decisions of individuals. Along the BGP of the overlapping generations version of the Romer (1990) model, the parameters have to be such that the birth rate equals *μ*, whereas in the overlapping generations version of the Jones (1995a) model, the parameters have to be such that the population grows at rate *n* = *β* − *μ*, where *β* > *μ* denotes the birth rate.^5^

In this stylized specification, decreases in fertility lead to both a slowdown of population growth *and* to population aging, while decreases in mortality *only* increase the population growth rate and have *no effect* on the aggregate age decomposition. To see this, consider the size of a particular cohort born at time *t*_0_ which reads $N(t_0,t) = \beta N(t_0) e^{-\mu(t-t_0)} = \beta N(0) e^{\beta t_0} e^{-\mu t}$ with *N*(0) being the initial population size. The proportionate age structure of the population, that is, the size of a particular cohort in relation to the whole population (cf. Preston et al. 2001, p. 144-147) is then given by

1 $$ \frac{N(t_0,t)}{N(t)} = \frac{\beta N(0) e^{\beta t_0} e^{-\mu t}}{N(0) e^{(\beta-\mu) t} } = \beta e^{-\beta(t-t_0)} . $$

Taking the derivative with respect to the birth rate leads to

2 $$ \frac{\partial \left[ N(t_0,t)/N(t) \right]}{\partial \beta} = e^{-\beta (t-t_0)} - \beta e^{-\beta (t-t_0)} (t-t_0), $$

where we see that a decrease in the birth rate decreases the proportionate age structure for young cohorts (whose age *t* − *t*_0_ is low), while the converse holds true for older cohorts. Consequently, a decrease in the birth rate raises the mean age of the population. Since *μ* does not show up in Eq. 1, changes in mortality do not impact the proportionate age structure and therefore a decrease in mortality has no effect on the mean age of a population. This hinges on the assumption that mortality is age-independent, which is required for analytical solvability of the model. By contrast, if old-age mortality could be reduced, while mortality for the rest of the population stayed constant, this would increase the mean age of the population.

In the Romer (1990) framework, demographic change can then be analyzed by contemporaneous proportional shifts in both fertility and mortality. In this context, proportional means “in the same order of magnitude,” such that a constant population level is preserved. Decreasing fertility and mortality therefore leads to population aging and leaves the population size constant. In the Jones (1995a) framework, demographic change can be analyzed by changing the birth rate and the mortality rate independently of each other. Decreases in the birth rate ceteris paribus lead to population aging and a slowdown in the population growth rate, while decreases in mortality ceteris paribus increase the population growth rate but have no effect on the age structure.

Finally, we want to make some assumptions explicit that are implied by the overlapping generations and R&D-based growth literature on which we base our analyses. These include that the labor supplies of different individuals, irrespective of their age, are perfect substitutes, individuals have perfect foresight, and they take the behavior of other agents as given, that is, they do not engage in strategic interactions. Furthermore, we abstract from bequests which would, in general, weaken the effects of generational turnover. However, unless full bequests were assumed (and therefore, the representative agent assumption would be reintroduced through the back door), our results would not change qualitatively.^6^

### Consumption side

Suppressing time subscripts, consider that a certain individual maximizes its discounted stream of lifetime utility

3 $$ u = \int_{t_0}^\infty e^{-(\rho+\mu)(\tau-t_0)} \left[ \log(c) + \gamma \log(\beta) \right] d \tau, $$

where *c* refers to individual consumption of the final good and *γ* ∈ (0, 1) is the weight of children. The subjective time discount rate *ρ* > 0 is augmented by the mortality rate *μ* > 0 because—as compared to the case of no lifetime uncertainty—individuals who face the risk of death are less likely to postpone consumption and fertility into the future. Note that the parameter restriction on *γ* ensures a strictly positive but finite birth rate, while the assumption of logarithmic utility is needed for analytical tractability.

We implement the assumption of Yaari (1965) that individuals insure themselves against the risk of dying with positive assets by using their savings to buy actuarial notes of a fair life insurance company. Such a fair life insurance company basically redistributes wealth of individuals who died among those who survived and therefore the real rate of return on capital is augmented by the mortality rate. Furthermore, we assume that the costs of children are represented by foregone consumption in the sense that parents provide their offspring with a certain fraction *ψ* > 0 of their own consumption. Hence, we follow a special case of Barro and Becker (1989), where costs of children are solely measured in consumption goods. This assumption allows us to derive a constant positive birth rate, that is, the model neither features the counter-factual cases of zero fertility or hyper-exponential population growth.^7^ Consequently, the wealth constraint of individuals reads

4 $$ \dot{k} = (r+\mu-\delta)k + \hat{w} - (1 + \psi \beta)c,$$

where *k* refers to the individual capital stock, *r* is the rental rate of capital, *δ* > 0 is the rate at which machines depreciate, and $\hat{w}$ represents noninterest income consisting of wage payments and possible lump-sum redistributions. Furthermore, we refer to final goods as numéraire. Note that we assume an inelastic labor supply, that is, each individual supplies all its available labor, disregarding the wage rate. The left-hand side of the constraint denotes the change in the individual’s capital stock, while the right-hand side consists of total individual savings, that is, capital income and noninterest income in excess of consumption expenditures. These consumption expenditures comprise consumption of the parent and consumption granted to children, with the latter representing a constant fraction *ψ* of total household consumption for each child.^8^ Carrying out utility maximization subject to the wealth constraint yields a constant birth rate

5 $$ \beta = \frac{\gamma}{(1-\gamma) \psi} $$

that increases in the desire of individuals toward having children and decreases in the costs of each child. This can easily be seen by taking the derivatives of Eq. 5 with respect to the two parameters *γ* and *ψ*. The population growth rate *n* = *γ*/((1 − *γ*)*ψ*) − *μ* is positive if the desire for having children is strong enough in relation to the costs of each child such that fertility exceeds mortality, it is negative if the converse holds true, and it is zero if *ψ* = *γ*/((1 − *γ*)*μ*). Since the birth rate is constant over time, we can derive the individual Euler equation

6 $$ \frac{\dot{c}}{c} = r-\delta-\rho , $$

stating that consumption expenditure growth is positive if and only if the interest rate *r* − *δ* exceeds the discount rate *ρ*. However, our economy does not feature only one single representative individual, and we have to use certain aggregation rules to come up with expressions for aggregate consumption expenditure growth as well as laws of motion for aggregate capital. This is done in Section 2.2.1 for the BGP of the Romer (1990) model, which requires a constant population, and in Section 2.2.2 for the BGP of the Jones (1995a) model, which requires a growing population. In Section 3.3, we compare the implications of demographic change within all possible combinations of the demographic outcomes and the two underlying growth frameworks.

#### Aggregation in case of a constant population

In our framework, agents are heterogeneous with respect to age and therefore also with respect to accumulated wealth because older agents have had more time to build up positive assets in the past. In order to get to the law of motion for aggregate capital and to the economy-wide (“aggregate”) Euler equation, we have to apply the following rules to integrate over all cohorts alive at time *t* (cf. Heijdra and van der Ploeg 2002)

7 $$ K(t)\equiv \int_{-\infty}^{t}k(t_0,t)N(t_0,t)dt_0, $$

8 $$ C(t)\equiv \int_{-\infty}^{t}c(t_0,t)N(t_0,t)dt_0, $$

where we denote aggregate variables by uppercase letters. After applying our demographic assumptions for the BGP of the Romer (1990) case, we can rewrite these rules as follows:

9 $$ C(t) \equiv \mu N \int_{-\infty}^{t}c(t_0,t)e^{\mu(t_0-t)}dt_0, $$

10 $$ K(t) \equiv \mu N \int_{-\infty}^{t}k(t_0,t)e^{\mu(t_0-t)}dt_0 $$

because $\psi=\gamma/((1-\gamma)\mu) \Rightarrow \beta=\mu$ in case of a constant population size *N*, and each cohort is of size $\mu N e^{\mu(t_0-t)}$ at a certain point in time *t* > *t*_0_. Consequently, we have that $\int_{-\infty}^{t} \mu N e^{\mu(t_0-t)}dt_0 = N $ holds for the total population size at time *t* and due to our assumption of inelastic labor supply also for the size of the workforce *L* ≡ *N*.^9^ After carrying out the calculations described in the Online-Appendix, we arrive at the following expressions for the law of motion of aggregate capital and for the aggregate Euler equation:

11 $$ \dot{K} = (r-\delta) K(t) - \left(1+\frac{\gamma}{1-\gamma}\right) C(t) + \hat{W}(t), $$

12 $$ \frac{\dot{C}(t)}{C(t)} = r-\rho-\delta - \frac{\gamma}{(1-\gamma)\psi} \frac{\rho+\gamma/((1-\gamma)\psi)}{1+ \gamma / (1-\gamma)}\frac{K(t)}{C(t)}, $$

where we have that $\left[(\rho+\gamma/((1-\gamma)\psi)) K(t))/((1+\gamma / (1-\gamma)) C(t)\right] = [C(t) - c(t,t)N] /C(t)$, which we denote by Ω. Note that average consumption in an economy is always higher than consumption of newborns because newborns do not have any accumulated capital yet. Therefore, aggregate consumption *C*(*t*), which can be written as the product of average consumption and the population size, is always higher than consumption of the newborns multiplied by the population size, *c*(*t*, *t*)*N*, and hence Ω ∈ (0, 1). Consequently, *aggregate* consumption expenditure growth will always be lower than *individual* consumption expenditure growth. The intuitive explanation is that at each instant, a fraction *μ* of older and therefore wealthier individuals die, and they are replaced by poorer newborns. Since the latter can afford less consumption than the former, the turnover of generations slows down aggregate consumption expenditure growth as compared to individual consumption expenditure growth (cf. Heijdra and van der Ploeg 2002). As regards the law of motion for aggregate capital, we see that the mortality rate does not show up. The reason is that the life insurance company only redistributes capital between cohorts and does not itself create or subtract capital from the economy as a whole.

#### Aggregation in case of a growing population

In case of the BGP of the Jones (1995a) model, population growth is positive. The aggregation rules in such a setting remain the same as in Section 2.2.1, but the demographic assumptions change because the period fertility rate exceeds the mortality rate *μ*. Therefore, the population grows at rate *n* = *γ*/((1 − *γ*)*ψ*) − *μ*, and we normalize the initial population size to *N*(0), being equivalent to the initial workforce *L*(0). We can then write the size of a cohort born at *t*_0_ < *t* at a certain point in time as

13 $$ N(t_0,t) = \frac{\gamma}{(1-\gamma) \psi} L(0) e^{\frac{\gamma}{(1-\gamma) \psi} t_0} e^{-\mu t}. $$

Integrating over all cohorts alive yields the population size, that is, the available amount of labor at time *t* as

14 $$ L(t) = L(0) e^{\left[ \frac{\gamma}{(1-\gamma) \psi} - \mu \right] t}. $$

Therefore, we can define the aggregate capital stock and aggregate consumption according to

15 $$C(t) \equiv \frac{\gamma}{(1-\gamma) \psi} L(0) e^{-\mu t} \int_{-\infty}^{t}c(t_0,t)e^{ \frac{\gamma}{(1-\gamma) \psi} t_0} \ dt_0, $$

16 $$ K(t) \equiv \frac{\gamma}{(1-\gamma) \psi} L(0) e^{-\mu t} \int_{-\infty}^{t}k(t_0,t)e^{ \frac{\gamma}{(1-\gamma) \psi} t_0} \ dt_0. $$

After carrying out the calculations described in the Online-Appendix, we arrive at the law of motion for aggregate capital and the aggregate Euler equation:

17 $$ \dot{K} = (r-\delta) K(t) - \left[1+ \frac{\gamma}{1-\gamma} \right] C(t) + \hat{W}(t), $$

18 $$ \frac{\dot{C}}{C} = r-\rho-\delta + \frac{\gamma}{(1-\gamma) \psi} \left[ \frac{H(t)}{K(t)+H(t)} \right] - \mu, $$

where *H*(*t*) refers to aggregate human wealth. If we denote *H*(*t*)/(*K*(*t*) + *H*(*t*)) by Ω′, we can immediately conclude that Ω′ ∈ (0, 1) holds, and economy-wide consumption expenditure growth differs from individual consumption expenditure growth. Now the argument still holds that an increase in mortality means that older and richer individuals die more frequently, and their replacement by newborns without capital leads to a slowdown of aggregate consumption expenditure growth as compared to individual consumption expenditure growth. However, there is now an additional effect arising from changes in fertility: a higher fertility rate *γ*/ ((1 − *γ*) *ψ*) leads to faster population growth which in turn spurs aggregate consumption expenditure growth. Note that the law of motion for aggregate capital is the same as in the case of a constant population size (cf. Buiter 1988).

### Production side

Now we turn to the production side of our model economies which closely follows Romer (1990) and Jones (1995a). The final goods sector produces the consumption aggregate with labor and intermediates as inputs. To have a sensible economic interpretation, we refer to intermediate varieties as differentiated machines. The production function of the final goods sector can be written as

19 $$ Y = L_Y^{1-\alpha} \int_0^{A} x_{i}^{\alpha} di ,$$

where *Y* represents output of the consumption aggregate, that is, the gross domestic product (GDP) of a country, *L*_*Y*_ refers to labor used in final goods production, *A* is the technological frontier (loosely speaking, the “number” of differentiated machines available), *x*_*i*_ is the amount of a certain specific machine *i* used in final goods production, and *α* ∈ (0, 1) is the intermediate input share. Profit maximization and the assumption of perfect competition in the final goods sector imply that factors are paid their marginal products

20 $$ w_Y = (1-\alpha) \frac{Y}{L_Y}, $$

21 $$ p_{i} = \alpha L_Y^{1-\alpha} x_{i}^{\alpha-1}, $$

where *w*_*Y*_ refers to the wage rate paid in the final goods sector and *p*_*i*_ to prices paid for intermediate inputs.

The intermediate goods sector is monopolistically competitive in the vein of Dixit and Stiglitz (1977) such that each firm produces one of the differentiated machines. In so doing, it has to purchase one machine-specific blueprint from the R&D sector and afterwards employ capital as variable input in production. The costs of blueprints represent fixed costs to each firm. Free entry ensures that operating profits equal fixed costs such that overall profits are zero.^10^ After an intermediate goods producer has purchased a blueprint, it can transform one unit of capital into one unit of the intermediate good, that is, *k*_*i*_ = *x*_*i*_. Thus, operating profits can be written as

22 $$ \begin{array}{rll} \pi_i & = & p_i k_i - r k_i \\ & = & \alpha L_Y^{1-\alpha} k_i^{\alpha} - r k_i . \end{array} $$

Profit maximization of firms then yields prices of machines

23 $$ p_i = \frac{r}{\alpha} , $$

where 1/*α* is the markup over marginal cost (cf. Dixit and Stiglitz 1977). Note that this holds for all firms, so we can drop the index *i* from now on. The aggregate capital stock is equal to the amount of all intermediates produced, that is, *K* = *A* *x*, such that Eq. 19 becomes

24 $$ Y = (A L_Y)^{1-\alpha} K^\alpha $$

and technological progress appears as labor augmenting.

The R&D sector employs scientists to discover new blueprints. Depending on the productivity of scientists, *λ*, and the size of technology spillovers, *ϕ*, the number of blueprints evolves according to

25 $$ \dot{A} = \lambda A^\phi L_A , $$

where *L*_*A*_ denotes the amount of scientists employed. We see that the technological frontier expands faster if scientists are more productive or technological spillovers are higher.^11^ If *ϕ* = 1, spillovers are strong enough such that developing a constant fraction of new blueprints does not become ever more difficult with an expanding technological frontier. If, by contrast, *ϕ* < 1, the spillovers are insufficiently low and developing a constant fraction of new blueprints becomes more and more difficult with increasing *A*.^12^ In the former case, our economy follows the Romer (1990) scenario; in the latter case, it follows the Jones (1995a) scenario. Furthermore, there is perfect competition in the R&D sector, and research firms maximize their profits *π*_*A*_

26 $$ \max\limits_{L_A} \ \pi_A = p_A \lambda A^\phi L_A - w_A L_A, $$

with *p*_*A*_ representing the price of a blueprint. The first order condition pins down wages in the research sector to

27 $$ w_A = p_A \lambda A^\phi . $$

The interpretation of this equation is straightforward: wages of scientists increase in their productivity as well as in the price that research firms can charge for blueprints. If *ϕ* = 1, an expanding technological frontier gradually increases wages of scientists, whereas *ϕ* < 1 means that the increases in scientist’s wages caused by technological progress become smaller and smaller. Since the wages of workers in the final goods sector linearly increase in *A*, the latter implies that being a scientist would become less and less attractive.

### Market clearing

There is perfect labor mobility between sectors, therefore wages of final goods producers and wages of scientists equalize. The reason is that workers in the final goods sector and scientists are neither different with respect to education nor with respect to productivity. Consequently, if wages were higher in one of these two sectors, it would attract workers from the other sector until wages were equal again. Therefore, we can insert Eq. 20 into Eq. 27 to get to the following equilibrium condition

28 $$ p_A \lambda A^\phi = (1-\alpha) \frac{Y}{L_Y}. $$

Firms in the R&D sector can charge prices for blueprints that are equal to the present value of operating profits in the intermediate goods sector because there is always a potential entrant who is willing to pay that price due to free entry. Therefore, we have

29 $$ p_A = \int_{t}^\infty e^{-R(\tau)} \pi \ d\tau, $$

where $R(\tau) = \int_t^{\tau}(r(s) - \delta) \ ds$ such that the discount rate is the market interest rate. Via the Leibniz rule and the fact that prices of blueprints and therefore also the interest rate do not change along a BGP, we can obtain

30 $$ p_A = \frac{\pi}{r - \delta}. $$

This means that prices of blueprints are equal to operating profits of intermediate goods producers divided by the market interest rate. Note that this expression can only be obtained analytically for a constant interest rate and constant prices of blueprints, that is, along a BGP. Transitional dynamics cannot be analyzed analytically in such a framework (see Acemoglu 2009, p. 439-440 for a proof).^13^ Comparative statistics analyses should therefore be viewed as the comparison between different BGPs. Next, we obtain operating profits by using Eq. 22 as

31 $$ \pi = (1-\alpha) \alpha \frac{Y}{A} $$

such that Eq. 30 becomes

32 $$ p_A = \frac{(1-\alpha) \alpha Y}{(r-\delta) A}. $$

Assuming that labor markets clear, that is, *L* = *L*_*A*_ + *L*_*Y*_ , we can determine the amount of labor employed in the final goods sector and in the R&D sector by using Eq. 28:

33 $$ \begin{array}{rll} L_Y & = & \frac{(r-\delta) A^{1-\phi}} {\alpha \lambda}, \\ L_A & = & L - \frac{(r-\delta) A^{1-\phi}} {\alpha \lambda} . \end{array} $$

The interpretation of these two equations is straightforward: the higher the market interest rate on capital, *r* − *δ*, the higher are the opportunity costs of R&D investments and consequently, the lower is the number of scientists in the R&D sector and the larger is the number of workers in the final goods sector; the higher the productivity of researchers, *λ*, the more scientists in the R&D sector and the less workers in the final goods sector are employed; if knowledge spillovers *ϕ* are insufficiently low to prevent exponential growth of blueprints from becoming ever more difficult to achieve, an expanding technological frontier *A* reduces employment of scientists in the R&D sector and increases employment of workers in the final goods sector; finally, an increase in the intermediate share of final output, *α*, increases the number of scientists in the R&D sector and decreases the number of workers in the final goods sector because production of final output becomes less labor intensive. Inserting Eq. 33 into Eq. 25 and taking into account that blueprints once developed do not wear off leads to the evolution of technology as

34 $$ \dot{A} = \max \left\{ \lambda A^\phi L - \frac{(r-\delta) A} {\alpha} \ , \ 0 \right\}. $$

We see that the technological frontier expends faster, the larger the population size is. All factors identified above to reduce the amount of scientists employed in the R&D sector also reduce the pace of technological progress.

## Effects of demographic change on economic growth

From now on, we have to distinguish between the Romer (1990) case, where technological spillovers are strong and the population size is constant, and the Jones (1995a) case, where technological spillovers are weaker and the population grows at rate *n*. We derive the per capita growth rates along the BGPs in these cases and analyze their dependence upon demographic change. In the next two subsections, we focus on interior solutions, where growth rates are positive. When we summarize our results in Section 3.3, we will, however, also consider the boundary solutions.

### The BGP growth rate in the Romer (1990) case

After implementing the central assumption *ϕ* = 1 of the Romer (1990) model, the growth rate of the economy can be written as

35 $$ g = \lambda L - \frac{r-\delta } {\alpha} $$

because we know that along a BGP, we have $\dot{A}/A = \dot{C}/C = \dot{K}/K = g$. To eliminate the endogenous market rate of return on capital, we use the aggregate Euler equation for a constant population size and get the following expression:

36 $$ r = g + \rho + \delta + \mu (\rho+\mu)\frac{K}{C}. $$

In contrast to a setting with a representative infinitely lived agent, there is still an unknown expression to account for, namely *K*/*C*. Therefore, we rewrite the law of motion of aggregate capital in our closed economy as $\dot{K} = Y - C - \delta K$ such that we get the additional equation

37 $$ g = \frac{r}{\alpha^2} - \frac{C}{K} - \delta, $$

where we used that *Y*/*K* = *r*/*α* ^2^. Altogether, we therefore have the three Eqs. 35, 36, and 37 to solve for the three unknowns *g*, *r*, and *ξ* = *C*/*K*.^14^ The associated BGP growth rate of the economy boils down to

38 $$ \begin{array}{rll} g^{BGP}_R & = & \frac{\alpha (L (1+\alpha) \lambda -\rho ) -\left(\alpha ^2-1\right) \delta } {2 \alpha (1+\alpha)} \\ & + & \frac{\sqrt{(\gamma -1)^2 ((\alpha -1) (\alpha \delta +\delta +L \alpha \lambda )-\alpha \rho )^2 \psi ^4+4 \alpha ^3 \gamma (\gamma +\rho \psi -\rho \psi \gamma ) \psi ^2}}{2 \alpha (1+\alpha) (\gamma -1) \psi ^2}, \end{array} $$

where the subscript refers to the Romer (1990) case, and we used *μ* = *β* = *γ*/((1 − *γ*)*ψ*). Recall that the appropriate interpretation of the assessment of parameter changes is that two different BGPs are compared to each other. Now, we can state the first central result.

#### **Proposition 1**

*In case of the endogenous growth in the spirit of Romer (* 1990 *), increasing longevity has a positive effect on the BGP growth rate of an economy.*

#### Proof

The derivatives of Eq. 38 with respect to *γ* and *ψ* are

$$ \frac{\partial g_{R}^{BGP}}{\partial \gamma } = \frac{\alpha ^2 ((\gamma -1) \rho \psi -2 \gamma )}{(1+\alpha) (\gamma -1)^2 \chi}, \quad \frac{\partial g_{R}^{BGP}}{\partial \psi } = \frac{\alpha ^2 \gamma ((\gamma -1) \rho \psi -2 \gamma )}{(1+\alpha) (\gamma -1) \psi \chi}, $$

with

$$ \chi = \sqrt{(\gamma -1)^2 ((\alpha -1) (\alpha \delta +\delta +L \alpha \lambda )-\alpha \rho )^2 \psi ^4+4 \alpha ^3 \gamma (\gamma +\rho \psi -\rho \psi \gamma ) \psi ^2}. $$

Since we know that *δ*, *ρ*, and *λ* are positive and that *α*, *γ* ∈ (0, 1), we immediately see that *χ* is positive. Furthermore, the nominator is negative in both $\partial g_{R}^{BGP} / \partial \gamma$ and $\partial g_{R}^{BGP} / \partial \psi$, while the denominator is positive in $\partial g_{R}^{BGP} / \partial \gamma$ and negative in $\partial g_{R}^{BGP} / \partial \psi$. As a consequence, the BGP growth rate decreases in *γ* and increases in *ψ*. Since longevity decreases in *γ* and increases in *ψ*, the proposition holds. □

The intuition for this finding is that a decrease in mortality slows down the turnover of generations, and so a lower market interest rate is required to sustain a given growth rate of aggregate consumption expenditures. Due to the fact that future profits of R&D investments are discounted with this market interest rate, the profitability of R&D investments rises. Consequently, R&D efforts increase which fosters long-run growth because intertemporal knowledge spillovers in the Romer (1990) case are large enough for the effect to be sustainable.

### The BGP growth rate in the Jones (1995a) case

To get the BGP growth rate in the Jones (1995a) case denoted as $g^{BGP}_J $, we search for an expression where the growth rate of technology is constant and carry out the associated calculations in the Online-Appendix. This leads us to

39 $$ g^{BGP}_J = \frac{\gamma / ((1-\gamma) \psi) - \mu}{1-\phi} $$

and therefore we can state the second central result.

#### **Proposition 2**

*In the case of semi-endogenous growth in the spirit of Jones (* 1995a *), decreasing mortality raises the BGP growth rate of an economy, while decreasing fertility lowers it.*

#### Proof

The derivatives of Eq. 38 with respect to mortality and the parameters determining fertility are

$$ \begin{array}{rll} \frac{\partial g^{BGP}_J}{\partial \mu} &=& -\frac{1}{1-\phi}, \quad \frac{\partial g^{BGP}_J}{\partial \gamma} = \frac{\psi}{\left[(1-\gamma) \psi\right]^2(1-\phi)}, \\ \frac{\partial g^{BGP}_J}{\partial \psi} &=& -\frac{\gamma(1-\gamma)}{\left[(1-\gamma) \psi\right]^2(1-\phi)}. \end{array} $$

Recalling the parameter restrictions *ϕ* < 1, *γ* ∈ (0, 1) and *ψ* > 0, we see that the first and the third equation are always negative, while the second equation is always positive. Since fertility decreases in *ψ* and increases in *γ*, the proposition holds. □

The interpretation for this finding is that a decrease in mortality, holding fertility constant, and an increase in fertility, holding mortality constant, both increase the population growth rate. This leads to a permanent increase in the flow of scientists into the R&D sector. Consequently, a faster growth rate of the number of patents can be sustained.

### Comparison between the different frameworks

We start this subsection with a short summary regarding the possible effects of different demographic scenarios on long-run economic prosperity within the two different R&D-based growth frameworks.

#### Remark 1

Depending on the population growth rate and the extent of intertemporal knowledge spillovers, the following situations can occur:

1. If *γ*/((1 − *γ*) *ψ*) < *μ*, population growth is negative and no long-run BGP exists in both the Romer (1990) and the Jones (1995a) frameworks, that is, for all possible values of *ϕ*. However, Eq. 34 would prevent per capita GDP growth from becoming negative despite the shrinking population.^15^
2. If *γ*/((1 − *γ*) *ψ*) = *μ*, the population stagnates and
  - Per capita GDP grows exponentially along the BGP in the Romer (1990) framework, that is, for *ϕ* = 1.
  - The economy stagnates in the long run of the Jones (1995a) framework, that is, for *ϕ* < 1.
3. If *γ*/((1 − *γ*) *ψ*) > *μ*, population growth is positive and
  - There is hyper-exponential per capita GDP growth in the Romer (1990) framework, that is, for *ϕ* = 1.^16^
  - Per capita GDP grows exponentially along the BGP in the Jones (1995a) framework, that is, for *ϕ* < 1.

Since the empirically relevant cases for modern knowledge-based economies are those featuring positive nonaccelerating economic growth (cf. Kaldor 1957; Jones 1995a; Acemoglu 2009), we focus on the two relevant scenarios (2a, 3b) when comparing the effects of population aging on economic prosperity.

Considering scenario 2a, that is, the Romer (1990) model with demography, a decrease in mortality is accompanied by a proportional decrease in fertility. Both effects offset each other with regards to population growth such that the population size stays constant. Proposition 1 then allows us to conclude that the benefits of decreasing mortality for economic growth overcompensate the drawbacks of decreasing fertility. The intuitive explanation is that decreasing mortality also decreases the market interest rate by which future profits of R&D investments are discounted. This leads to a shift of resources to R&D and consequently fosters per capita output growth. The reason for the growth effect to be long-lasting is that intertemporal knowledge spillovers are strong. By contrast, a contemporaneous proportional decrease in fertility and mortality does not change the growth rate along a BGP in scenario 3b, that is, in the Jones (1995a) model with demography, as evident from Eq. 39. The reason is that intertemporal knowledge spillovers are too weak for a one-time resource shift to have long-lasting effects. We summarize this in the following remark:

#### Remark 2

In the case of endogenous growth in the spirit of Romer (1990), the benefits of decreasing mortality overcompensate for the drawbacks of proportional decreases in fertility with regards to long-run economic growth perspectives, while in case of semi-endogenous growth in the spirit of Jones (1995a), the benefits and drawbacks exactly offset each other.

Finally, we know that population aging is described by contemporaneous proportional decreases in fertility and mortality in scenario 2a (the Romer 1990 model with demography), whereas in scenario 3b (the Jones 1995a model with demography), it can be triggered by decreases in fertility only. Therefore, population aging has a positive impact on long-run economic growth if endogenous growth models are the accurate description of underlying growth processes, while it has a negative impact in semi-endogenous growth models as long as the fall in birth rates is not (over)-compensated by (more than) proportional exogenous decreases in mortality.^17^ We summarize this finding in the following proposition:

#### **Proposition 3**

*In the case of endogenous growth in the spirit of Romer (* 1990 *), population aging has a positive impact on the long-run economic growth rate.*

*In case of semi-endogenous growth in the spirit of Jones (* 1995a *), the following holds:*

- *If mortality is constant or decreases less than proportional to fertility, population aging negatively impacts long-run economic growth.*
- *If mortality declines proportional to fertility, population aging has no effect on long-run economic growth.*
- *If mortality declines more than proportional to fertility, population aging positively impacts long-run economic growth.*

#### Proof

This follows from Propositions 1 and 2 and the fact that population aging is represented by a decrease in *μ* in the Romer (1990) model and by a decrease in *β* in the Jones (1995a) model.^18^ However, a decrease in fertility whose effect on the population growth rate is fully compensated by proportional decreases in mortality has no growth effect in the Jones (1995a) case as evident from Eq. 39. In case that the fertility decline is associated with a more than proportional decline in mortality, population growth and long-run economic growth even accelerate. □

Altogether, we have been able to describe some important effects of demographic change on economic development within the realms of R&D-based economic growth models. In general, decreases in fertility negatively affect long-run growth, whereas decreasing mortality fosters long-run growth. In case of the Romer (1990) framework, the positive effects of decreasing mortality overcompensate for the negative effects of decreasing fertility, while in the Jones (1995a) framework, the positive and negative effects of contemporaneous proportional declines in both fertility and mortality offset each other. Furthermore, we have been able to show that the effects of population aging crucially depend on the underlying model used to describe the growth process. While population aging is in general beneficial in the Romer (1990) environment, the effect in the Jones (1995a) environment also depends on the relative change between fertility and mortality. If declining fertility is not fully compensated by declining mortality, population aging has a negative effect on economic growth, while the converse holds true if the mortality rate falls even stronger than fertility. In case that both mortality and fertility decrease proportionally, then the long-run economic growth rate is not affected at all.

## Conclusions

We set up a model of endogenous technological change that nests the Romer (1990) and the Jones (1995a) frameworks. We generalized this model by introducing finite individual planning horizons and thereby allowing for overlapping generations and age-specific heterogeneity of individuals. Furthermore, we introduced endogenous fertility decisions of individuals such that they care for the number of kids they have, while taking into account the associated costs. Altogether, we showed that the underlying demographic processes play a crucial role in describing the R&D intensity and thereby long-run economic growth perspectives of industrialized countries.

Our results regarding the effects of demographic change on long-run economic growth perspectives have been the following: (a) decreasing mortality positively affects long-run growth, (b) decreasing fertility negatively affects long-run growth, (c) the negative effects of decreases in fertility are overcompensated by the positive effects of decreases in mortality in the case of the Romer (1990) model, while they exactly offset each other in the Jones (1995a) framework, (d) population aging is beneficial for long-run economic growth in the Romer (1990) case, whereas it depends on the relative change between fertility and mortality whether it is associated with increasing or decreasing long-run economic growth in the Jones (1995a) case.

Our main conclusion is that currently ongoing demographic changes do not necessarily hamper technological progress and therefore economic prosperity. Simultaneously decreasing birth and death rates can even lead to an increase in the economic growth rate. These results, while holding in a stylized theoretical modeling framework, are also in line with empirical studies claiming that the negative effects of population aging on economic prosperity might not be as severe as often argued (cf. Bloom et al. 2008, 2010a, b).

In our framework, we relied on the notion of horizontal innovations. However, the results would carry over to endogenous and semi-endogenous growth models with vertical innovations (Grossman and Helpman 1991; Aghion and Howitt 1992; Segerström 1998) because the mechanisms causing economic growth in these models are very similar to those of the underlying models we used. It is, however, important to keep in mind that the welfare properties of models with horizontal innovation and of models with vertical innovation differ considerably. While faster economic growth is always beneficial in models with horizontal innovation, this is not true in models of vertical innovation. The reason is that—in the latter model class—introducing new products drives technologically inferior firms out of business. The associated negative welfare effect could potentially be strong enough to overcompensate for the positive welfare effects of technological progress through economic development. Frameworks that integrate horizontal and vertical innovation (cf. Young 1998; Peretto 1998; Dinopoulos and Thompson 1998) feature a balanced economic growth rate that positively depends on population growth *and* on the fraction of labor devoted to R&D. Therefore, elements of both cases that we considered would be present when introducing demography to such a framework. However, Jones (1999) showed that these models require very strong parameter restrictions for a balanced growth path to exist which limits their generality.

Of course we acknowledge that our modeling approach was only a first step toward a more thorough understanding of the impact of demographic change on long-run economic development as caused by purposeful R&D investments. In order to guarantee analytical solutions and to focus on the main channels by which demographic change can impact upon long-run economic growth, we abstracted from imperfect annuity markets, age-dependent mortality, educational decisions (and therefore human capital investments), and age-specific and occupation-specific heterogeneities of workers. We believe that these assumptions represent a sensible choice regarding the trade-off between analytical tractability and a concise exposition on the one hand and a detailed description of reality on the other hand. However, it makes clear that there is scope for further research.

## Electronic Supplementary Material

Below is the link to the electronic supplementary material.

(PDF 210 KB)

(TEX 15.9 KB)

## Footnotes

1 There are various channels by which human capital accumulation can foster economic growth (see for example Lucas 1988; Galor and Weil 2000).

2 I would like to thank an anonymous referee for pointing out this issue.

3 For an interesting study of physical capital taxation and labor income taxation in an overlapping generation growth model, see Chin and Lai (2009). However, long-run growth in their framework is not driven by R&D investments.

4 I would like to thank Timo Trimborn for his helpful suggestions regarding the implementation of fertility decisions within these types of models.

5 Note that the period fertility rate is equivalent to the birth rate in such a demographic setting (cf. Preston et al. 2001, p. 93).

6 For an interesting study on the effects of accidental bequests on inequality and growth, see Miyazawa (2006).

7 An alternative specification would be to allow for time costs of children such that each child requires the parent to spend a fraction of time at home and therefore not supplying labor on the labor market (cf. Galor and Weil 2000). This would, however, lead to perpetually increasing fertility in our case. The reason is that the expression for the birth rate involves the ratio of individual consumption to wages, which grows in an overlapping generations framework, even along the BGP.

8 Note that we abstract from indivisibilities of children similar to the standard Barro and Becker (1989) formulation.

9 This implicitly assumes that each worker is endowed with one unit of human capital. For alternative specifications, see, for example, Eicher (1996) and Arnold (2002).

10 If positive overall profits were present, new firms would enter the market until these profits had vanished.

11 Note that we do not allow for the possibility of duplication in the research process for the sake of comparability between the Romer (1990) model and the Jones (1995a) model. However, allowing different researchers to develop the same blueprint would not change our results qualitatively.

12 This can easily be shown by dividing Eq. 25 by the technological frontier *A*.

13 See Schmidt (2003) for a numerical method.

14 We solved the system using Mathematica. The corresponding file is available upon request. Note that there are two solution triples for *g*, *r*, and *ξ*. One of them features a negative *ξ*, and therefore it can be ruled out by economic arguments because neither the aggregate capital stock nor aggregate consumption expenditures can become negative. We therefore restrict our attention to the economically meaningful solution triple.

15 Strictly interpreted, in this case and for *t* → ∞, the corresponding societies would become extinct.

16 This can be seen by investigating Eqs. 35 and 38.

17 Recall that proportional means “in the same order of magnitude”, such that a constant population level is preserved.

18 Note that changes in the demographic structures are either triggered by changes in *γ* or by changes in *ψ*.
