# Supplementary material for: Population aging and endogenous economic growth
Source: J Popul Econ. 2012 Sep 15;26(2):811–34. doi: 10.1007/s00148-012-0441-9 (PMC3617593; doi:10.1007/s00148-012-0441-9)
Supplement: Supplementary file 1 — (PDF 210 KB) [file 148_2012_441_MOESM1_ESM.pdf]

Population aging and endogenous economic growth

Klaus Prettner

Online Appendix

The individual Euler equation: The current value Hamiltonian is

$$H = \log(c) + \gamma \log(\beta) + \lambda \left[(r + \mu - \delta)k + \hat{w} - (1 + \psi\beta)c \right].$$

The first order conditions are

$$\frac{1}{c} = \lambda(1 + \psi\beta) \quad \text{(A.1)}$$

$$\frac{\gamma}{\beta} = \lambda\psi c \quad \text{(A.2)}$$

$$\dot{\lambda} = (\rho + \delta - r)\lambda. \quad \text{(A.3)}$$

From equation (A.2) the birth rate follows as

$$\beta = \frac{\gamma}{(1-\gamma)\psi}. \quad \text{(A.4)}$$

Taking the time derivative of equation (A.1) and plugging it into equation (A.3) yields

$$\frac{\dot{c}}{c} = r - \rho - \delta$$

which is the familiar individual Euler equation.

Aggregate capital and aggregate consumption in the Romer (1990) case: Note that we set $\beta \equiv \mu$ in this case. Following Heijdra and van der Ploeg (2002) and differentiating aggregate consumption and aggregate capital with respect to time yields

$$\begin{aligned}
\dot{C}(t) &= \mu N \left[\int_{-\infty}^{t} \dot{c}(t_0, t) e^{\mu(t_0 - t)} dt_0 - \mu \int_{-\infty}^{t} c(t_0, t) e^{\mu(t_0 - t)} dt_0 \right] + \mu N c(t, t) - 0 \\
&= \mu N c(t, t) - \mu C(t) + \mu N \int_{-\infty}^{t} \dot{c}(t_0, t) e^{-\mu(t - t_0)} dt_0, \qquad \text{(A.5)} \\
\dot{K}(t) &= \mu N \left[\int_{-\infty}^{t} \dot{k}(t_0, t) e^{\mu(t_0 - t)} dt_0 - \mu \int_{-\infty}^{t} k(t_0, t) e^{\mu(t_0 - t)} dt_0 \right] + \mu N k(t, t) - 0 \\
&= \mu N \underbrace{k(t, t)}_{=0} - \mu K(t) + \mu N \int_{-\infty}^{t} \dot{k}(t_0, t) e^{-\mu(t - t_0)} dt_0. \qquad \text{(A.6)}
\end{aligned}$$

From the wealth constraint of an individual it follows that

$$\begin{aligned}
\dot{K}(t) &= -\mu K(t) + \mu N \int_{-\infty}^{t} \left[(r+\mu-\delta)k(t_0,t) + \hat{w}(t) - (1+\psi\mu)c(t_0,t)\right] e^{-\mu(t-t_0)} dt_0 \\
&= -\mu K(t) + (r+\mu-\delta)\mu N \int_{-\infty}^{t} k(t_0,t) e^{-\mu(t-t_0)} dt_0 \\
&\quad -\mu(1+\psi\mu)N \int_{-\infty}^{t} c(t_0,t) e^{-\mu(t-t_0)} dt_0 + N \left(\frac{\mu \hat{w} e^{-\mu(t-t_0)}}{\mu} \right)_{-\infty}^{t} \\
&= -\mu K(t) + (r+\mu-\delta)K(t) - (1+\psi\mu)C(t) + \hat{W}(t) \\
&= (r-\delta)K(t) - (1+\psi\mu)C(t) + \hat{W}(t) \\
&= (r-\delta)K(t) - \left(1 + \frac{\gamma}{1-\gamma}\right) C(t) + \hat{W}(t)
\end{aligned}$$

which is the law of motion for aggregate capital. In the last line we used that $\mu = \gamma/((1-\gamma)\psi)$ in the Romer (1990) case. Reformulating an agent's optimization problem subject to its lifetime budget restriction, stating that the present value of lifetime consumption expenditures have to be equal to the present value of lifetime non-interest income plus initial assets, yields the optimization problem

$$\begin{aligned}
\max_{c(t_0,\tau)} \quad U &= \int_{t}^{\infty} e^{(\rho+\mu)(t-\tau)} \left[\log(c(t_0,\tau)) + \gamma \log(\mu)\right] d\tau \\
s.t. \qquad & k(t_0,t) + \int_{t}^{\infty} \hat{w}(\tau) e^{-R^A(t,\tau)} d\tau = (1+\psi\mu) \int_{t}^{\infty} c(t_0,\tau) e^{-R^A(t,\tau)} d\tau,
\end{aligned} \tag{A.7}$$

where $R^A(t,\tau) = \int_t^\tau (r(s)+\mu-\delta) ds$. The FOC to this optimization problem is

$$\frac{1}{c(t_0,\tau)} e^{(\rho+\mu)(t-\tau)} = \lambda(t)(1+\psi\mu) e^{-R^A(t,\tau)}.$$

In period $(\tau = t)$ we have

$$c(t_0,t) = \frac{1}{\lambda(t)(1+\psi\mu)}.$$

Therefore we can write

$$\begin{aligned}
\frac{1}{c(t_0,\tau)} e^{(\rho+\mu)(t-\tau)} &= \frac{1}{c(t_0,t)} e^{-R^A(t,\tau)} \\
c(t_0,t) e^{(\rho+\mu)(t-\tau)} &= c(t_0,\tau) e^{-R^A(t,\tau)}.
\end{aligned}$$

Integrating and using equation (A.7) yields

$$\begin{aligned}
\int_{t}^{\infty} c(t_0,t) e^{(\rho+\mu)(t-\tau)} d\tau &= \int_{t}^{\infty} c(t_0,\tau) e^{-R^A(t,\tau)} d\tau \\
\frac{c(t_0,t)}{\rho+\mu} \left[-e^{(\rho+\mu)(t-\tau)}\right]_{t}^{\infty} &= \frac{1}{1+\psi\mu} \left[k(t_0,t) + \int_{t}^{\infty} \hat{w}(\tau) e^{-R^A(t,\tau)} d\tau\right] \\
\Rightarrow c(t_0,t) &= \frac{\rho+\mu}{1+\psi\mu} \left[k(t_0,t) + h(t)\right],
\end{aligned} \tag{A.8}$$

where $h \equiv \int_t^\infty \hat{w}(\tau)e^{-R^A(t,\tau)}d\tau$ refers to human wealth, that is, non-interest wealth of individuals. Human wealth does not depend on the date of birth because productivity and lump-sum transfers are age independent. The above calculations show that optimal consumption in the planning period is proportional to total wealth with a marginal propensity to consume of $(\rho+\mu)/(1+\psi\mu)$. Aggregate consumption evolves according to

$$\begin{aligned} C(t) &\equiv \mu N \int_{-\infty}^{t} c(t_0,t)e^{\mu(t_0-t)}dt_0 \\ &= \mu N \int_{-\infty}^{t} e^{\mu(t_0-t)}\frac{\rho+\mu}{1+\psi\mu}\left[k(t_0,t)+h(t)\right]dt_0 \\ &= \frac{\rho+\mu}{1+\psi\mu}\left[K(t)+H(t)\right], \end{aligned} \tag{A.9}$$

where $H(t) = Nh(t)$ is aggregate human wealth. Note that newborns do not own capital because there are no bequests. Therefore

$$c(t,t) = \frac{\rho+\mu}{1+\psi\mu}h(t) \tag{A.10}$$

holds for each newborn individual. Putting equations (A.5), (A.9), (A.10) and the individual Euler equation together yields

$$\begin{aligned} \dot{C}(t) &= \mu\frac{\rho+\mu}{1+\psi\mu}H(t) - \mu\frac{\rho+\mu}{1+\psi\mu}\left[K(t)+H(t)\right] + \\ &\quad \mu N \int_{-\infty}^{t} (r-\rho-\delta)c(t_0,t)e^{-\mu(t-t_0)}dt_0 \\ &= \mu\frac{\rho+\mu}{1+\psi\mu}H(t) - \mu\frac{\rho+\mu}{1+\psi\mu}\left[K(t)+H(t)\right] + (r-\rho-\delta)C(t) \\ \Rightarrow \frac{\dot{C}(t)}{C(t)} &= r-\rho-\delta-\mu\frac{\rho+\mu}{1+\psi\mu}\frac{K(t)}{C(t)} \\ &= r-\rho-\delta-\frac{\gamma}{(1-\gamma)\psi}\frac{\rho+\gamma/((1-\gamma)\psi)}{1+\gamma/(1-\gamma)}\frac{K(t)}{C(t)} \\ &= r-\rho-\delta-\frac{\gamma}{(1-\gamma)\psi}\underbrace{\frac{C(t)-c(t,t)N}{C(t)}}_{\in(0,1)} \end{aligned}$$

which is the aggregate Euler equation that differs from the individual Euler equation by the term $-\mu\left[C(t)-c(t,t)N\right]/C(t)$.

Aggregate capital and aggregate consumption in the Jones (1995a) case: Using our demographic assumptions for the Jones (1995a) case, we can write the size of a cohort

born at $t_0 < t$ at time t as

$$\begin{aligned} N(t_0,t) &= \beta L(t_0)e^{-\mu(t-t_0)} \\ &= \beta L(0)e^{nt_0}e^{-\mu(t-t_0)} \\ &= \beta L(0)e^{\beta t_0}e^{-\mu t}. \end{aligned}$$

Integrating over all cohorts yields the population size as

$$\begin{aligned} L(t) &= \int_{-\infty}^{t} \beta L(0)e^{\beta t_0}e^{-\mu t}dt_0 \\ &= L(0)e^{(\beta-\mu)t}. \end{aligned}$$

Following Buiter (1988) and differentiating aggregate consumption and aggregate capital with respect to time yields:

$$\begin{aligned} \dot{C}(t) &= \left[\int_{-\infty}^{t} \beta L(0)e^{-\mu t}\dot{c}(t_0,t)e^{\beta(t_0)} - \mu\beta L(0)e^{-\mu t}c(t_0,t)e^{\beta t_0}dt_0\right] \\ &+ \beta L(0)e^{-\mu t}c(t,t)e^{\beta t} - 0 \\ &= \beta L(0)e^{-\mu t}c(t,t)e^{\beta t} - \mu C(t) + \beta L(0)e^{-\mu t}\int_{-\infty}^{t} \dot{c}(t_0,t)e^{\beta t_0}dt_0, \end{aligned} \tag{A.11}$$

$$\begin{aligned} \dot{K}(t) &= \left[\int_{-\infty}^{t} \beta L(0)e^{-\mu t}\dot{k}(t_0,t)e^{\beta(t_0)} - \mu\beta L(0)e^{-\mu t}k(t_0,t)e^{\beta t_0}dt_0\right] \\ &+ \beta L(0)e^{-\mu t}k(t,t)e^{\beta t} - 0 \\ &= \beta L(0)e^{-\mu t}\underbrace{k(t,t)}_{=0}e^{\beta t} - \mu K(t) + \beta L(0)e^{-\mu t}\int_{-\infty}^{t} \dot{k}(t_0,t)e^{\beta t_0}dt_0. \end{aligned} \tag{A.12}$$

From the individual wealth constraint it follows that

$$\begin{aligned} \dot{K}(t) &= -\mu K(t) + \beta L(0)e^{-\mu t}\int_{-\infty}^{t} \left[(r+\mu-\delta)k(t_0,t) + \hat{w}(t) - (1+\psi\beta)c(t_0,t)\right]e^{\beta t_0}dt_0 \\ &= -\mu K(t) + (r+\mu-\delta)\beta L(0)e^{-\mu t}\int_{-\infty}^{t} k(t_0,t)e^{\beta t_0}dt_0 \\ &\quad -\beta(1+\psi\beta)L(0)e^{-\mu t}\int_{-\infty}^{t} c(t_0,t)e^{\beta t_0}dt_0 + L(0)e^{-\mu t}\left(\frac{\beta\hat{w}(t)e^{\beta t_0}}{\beta}\right)_{-\infty}^{t} \\ &= -\mu K(t) + (r+\mu-\delta)K(t) - (1+\psi\beta)C(t) + \hat{W}(t) \\ &= (r-\delta)K(t) - (1+\psi\beta)C(t) + \hat{W}(t) \end{aligned}$$

which is the law of motion for aggregate capital. Note that the definition of aggregate non-interest income is $\hat{W}(t) = L(0)\hat{w}(t)e^{\beta-\mu}$. By making use of equation (A.8) for $\beta \neq \mu$,

we can write aggregate consumption as

$$\begin{aligned}
C(t) &\equiv \beta L(0)e^{-\mu t}\int_{-\infty}^{t} c(t_0,t)e^{\beta t_0}dt_0 \\
&= \beta L(0)e^{-\mu t}\int_{-\infty}^{t} e^{\beta t_0}\frac{\rho+\mu}{1+\psi\beta}\left[k(t_0,t)+h(t)\right]dt_0 \\
&= \frac{\rho+\mu}{1+\psi\beta}K(t)+\beta L(0)e^{-\mu t}\frac{\rho+\mu}{1+\psi\beta}\int_{-\infty}^{t} e^{\beta t_0}h(t)dt_0 \\
&= \frac{\rho+\mu}{1+\psi\beta}\left[K(t)+H(t)\right]. \qquad \text{(A.13)}
\end{aligned}$$

Note that the following definitions apply: $K(t) = \beta L(0)e^{-\mu t}\int_{-\infty}^{t} e^{\beta t_0}k(t_0,t)dt_0$ and $H(t) = L(0)e^{(\beta-\mu)t}h(t)$. Newborns do not own capital because there are no bequests, therefore

$$c(t,t) = \frac{\rho+\mu}{1+\psi\beta}h(t) \qquad \text{(A.14)}$$

holds for each newborn individual. Putting equations (A.11), (A.13), (A.14) and the individual Euler equation together yields

$$\begin{aligned}
\dot{C}(t) &= \beta\frac{\rho+\mu}{1+\psi\beta}H(t)-\mu\frac{\rho+\mu}{1+\psi\beta}\left[K(t)+H(t)\right]+ \\
&\quad \beta L(0)e^{-\mu t}\int_{-\infty}^{t}(r-\rho-\delta)c(t_0,t)e^{\beta t_0}dt_0 \\
&= \beta\frac{\rho+\mu}{1+\psi\beta}H(t)-\mu\frac{\rho+\mu}{1+\psi\beta}\left[K(t)+H(t)\right]+(r-\rho-\delta)C(t) \\
\Rightarrow \frac{\dot{C}(t)}{C(t)} &= r-\rho-\delta+\frac{\beta(\rho+\mu)H(t)-\mu(\rho+\mu)\left[K(t)+H(t)\right]}{(\rho+\mu)\left[K(t)+H(t)\right]} \\
&= r-\rho-\delta+\frac{\gamma}{(1-\gamma)\psi}\underbrace{\frac{H(t)}{K(t)+H(t)}}_{\Omega'\in(0,1)}-\mu
\end{aligned}$$

which is the aggregate Euler equation that differs from the individual Euler equation by the term $\gamma H(t)/\left[(K(t)+H(t))(1-\gamma)\psi\right]-\mu$. Note that we substituted optimal fertility decisions of households for β in the last line.

Operating profits for intermediate goods producers: Profits of intermediate goods producers can be rewritten as

$$\begin{aligned}
\pi &= \frac{r}{\alpha}x - rx \\
&= (1-\alpha)\alpha\frac{Y}{A}.
\end{aligned}$$

Labor input in both sectors: We determine the fraction of workers employed in the final goods sector and in the R&D sector by making use of the equilibrium condition (28) in the main text

$$\begin{aligned} p^A \lambda A^\phi &= (1-\alpha)\frac{Y}{L_Y} \\ L_Y &= \frac{(r-\delta)A^{1-\phi}}{\alpha\lambda} \\ \Rightarrow L_A &= L - \frac{(r-\delta)A^{1-\phi}}{\alpha\lambda}, \end{aligned}$$

where the last line follows from labor market clearing, that is, $L = L_A + L_Y$.

Rewriting production per capital unit: Production per capital unit can be written as a function of the interest rate and the intermediate share in final goods production

$$\begin{aligned} r &= \alpha p = \alpha^2 \frac{Y}{K}, \\ \Rightarrow \frac{Y}{K} &= \frac{r}{\alpha^2} \end{aligned} \tag{A.15}$$

The BGP growth rate in the Jones (1995a) case with demography: The growth rate of the economy is

$$g = \frac{\dot{A}}{A} = \frac{\lambda L_A}{A^{1-\phi}}.$$

Taking logarithms yields

$$\log g = \log(\lambda) + \log(L_A) - (1-\phi)\log(A).$$

Taking the derivative of this expression with respect to time and noting that along the BGP the growth rate is constant, yields

$$\begin{aligned} \frac{\dot{g}}{g} &= n - (1-\phi)g = 0 \\ \Rightarrow g &= \frac{n}{1-\phi} \\ &= \frac{\gamma/((1-\gamma)\psi) - \mu}{1-\phi}. \end{aligned}$$

References

Buiter WH (1988) Death, birth, productivity growth and debt neutrality. *The Economic Journal* 98(391):179–293.

Heijdra BJ, van der Ploeg F (2002) *Foundations of Modern Macroeconomics*. Oxford University Press. Oxford.

Jones CI (1995a) R&D-based models of economic growth. *Journal of Political Economy* 103(4):759–783.

Romer P (1990) Endogenous technological change. *Journal of Political Economy* 98(5):71–102.
